# Characterization of 843 children with Zika-related microcephaly in the first three years of life: An individual participant data meta-analysis of 12 cohorts in the Zika Brazilian Cohorts consortium

**DOI:** 10.1371/journal.pgph.0005425

**Published:** 2025-12-29

**Authors:** Demócrito de Barros Miranda-Filho, Ricardo Arraes de Alencar Ximenes, Ulisses Ramos Montarroyos, Marília Rosa Abtibol-Bernardino, Elizabeth B. Brickley, Celina Maria Turchi Martelli, Laura Cunha Rodrigues, Thália Velho Barreto de Araújo, Liana O. Ventura, Mariana Carvalho Leal, Darci Neves Santos, Letícia Marques dos Santos, Lucas Monteiro Santos, Mariana Rabelo Gomes, Isadora Cristina de Siqueira, Letícia Serra, Débora Patrícia Medeiros Santos Rios, Alessandra Carvalho, Antônio Moura Silva, Patrícia Silva Sousa, Marizélia Costa Ribeiro, Marcos Garcia Campos, Saulo Duarte Passos, Ana Paula Paschoalicchio Bertozzi, Rosa Estela Gazeta, Daniel T. Catalan, Ricardo Queiroz Gurgel, Aline de Siqueira Alves Lopes, Andrea Monteiro Correia Medeiros, Patrícia Brasil, Karin Nielsen-Saines, Zilton Vasconcelos, Andrea Araújo Zin, Marisa Márcia Mussi-Pinhata, Silvia Fabiana Biason de Moura Negrini, Bento Vidal de Moura Negrini, Carla Andrea Cardoso Tanuri Caldas, Daniela Vivacqua, Bernadete Perez Coelho, Lucíola de Fátima Albuquerque de Almeida Peixoto, Camila Bôtto-Menezes, Silvana Gomes Benzecry, Consuelo Silva de Oliveira, Joelma Karin Sagica Fernandes Paschoal, Emilene Monteiro Furtado Serra, Luna Thais Sousa Gomes, Maria Elisabeth Moreira, Cristina Barroso Hofer, on behalf of Zika Brazilian Cohorts Consortium (ZBC Consortium)

**Affiliations:** 1 Postgraduate Program in Health Sciences, University of Pernambuco, Recife, Pernambuco, Brazil,; 2 Postgraduate Program in Tropical Medicine, Federal University of Pernambuco, Recife, Pernambuco, Brazil,; 3 Postgraduate Program in Tropical Medicine, Tropical Medicine Foundation Doctor Heitor Vieira Dourado, Manaus, Amazonas, Brazil; 4 Amazon State University, Manaus, Amazonas, Brazil; 5 Department of Infectious Disease Epidemiology, London School of Hygiene & Tropical Medicine, London, United Kingdom; 6 Department of Collective Health, Aggeu Magalhaes Institute/Fiocruz, Recife, Pernambuco, Brazil; 7 Postgraduate Program in Collective Health, Federal University of Pernambuco, Recife, Pernambuco, Brazil; 8 Altino Ventura Foundation, Recife, Pernambuco, Brazil; 9 Pernambuco Eyes Hospital, Recife, Pernambuco, Brazil; 10 Medical Sciences Center, Postgraduate Program in Human Communication Health, Federal University of Pernambuco, Recife, Pernambuco, Brazil; 11 Institute of Collective Health, Federal University of Bahia, Salvador, Bahia, Brazil; 12 Laboratory of Investigation in Global Health and Neglected Diseases, Goncalo Moniz Institute – Fiocruz, Salvador, Bahia, Brazil; 13 Child Rehabilitation Center, SARAH Salvador Hospital, Salvador, Bahia, Brazil; 14 Department of Public Health, Federal University of Maranhão, São Luís, Maranhão, Brazil; 15 Reference Center on Neurodevelopment, Assistance and Rehabilitation of Children, São Luís, Maranhão, Brazil; 16 Department of Medicine, Federal University of Maranhão, São Luís, Maranhão, Brazil; 17 Pediatrics Department, Jundiaí Medical School, Jundiaí, São Paulo, Brazil; 18 Department of Medicine and Graduate Program in Health Sciences, Federal University of Sergipe, Aracaju, Sergipe, Brazil; 19 Department of Speech Therapy and Graduate Program in Health Sciences, Federal University of Sergipe, Aracaju, Sergipe, Brazil; 20 National Institute of Infectious Diseases/Fiocruz, Rio de Janeiro, Brazil; 21 Pediatric Infectious Disease, University of California, Los Angeles, California, United States of America; 22 Clinical Research Unit, Fernandes Figueira Institute/Fiocruz, Rio de Janeiro, Brazil; 23 Department of Pediatrics, Ribeirão Preto Medical School - University of São Paulo, Ribeirão Preto, São Paulo, Brazil; 24 Department of Neurosciences and Behavioral Sciences, Ribeirão Preto Medical School - University of São Paulo, Ribeirão Preto, São Paulo, Brazil; 25 Department of Infectious Diseases, Federal University of Rio de Janeiro, Rio de Janeiro, Brazil; 26 Department of Public Health of the Medical Sciences Center, Federal University of Pernambuco, Recife, Pernambuco, Brazil; 27 Department of Arbovirology and Hemorrhagic Fevers, Evandro Chagas Institute, Belém, Pará, Brazil; 28 Department of Neurology, Federal University of Pará Medical School, Belém, Pará, Brazil; Fundacao Oswaldo Cruz, BRAZIL

## Abstract

One of the main gaps in characterizing the congenital Zika syndrome (CZS) up to now is the small number of participants in individual studies. Pooling the data together overcomes the sample size-related limitations of individual studies and provides an opportunity for investigating heterogeneity between studies and, as appropriate, to better characterize the CZS**.** This study aims to describe adverse anthropometric, clinical, and neuroimaging outcomes in Brazilian children with Zika-related microcephaly.Individual participant data meta-analysis of 12 cohorts of the Zika Brazilian Cohorts Consortium (ZBC-Consortium), using primary data from children with Zika-related microcephaly, born between January/2015 and July/2018. We included 843 children with microcephaly at birth, at first evaluation, or over follow-up. Of 601 children with microcephaly at birth, 217 (36.1%) had moderate and 384 (63.9%) had severe microcephaly. Postnatal microcephaly occurred in 172 (20.4%) children. There was heterogeneity across studies. Severe microcephaly ranged from 11-87.5%; prematurity from 10-20% in most sites; low birth weight from 10-43.8%. Deficit of social attention, hyperreflexia, and persistence of primitive reflexes were reported in at least 50% of the children in the larger cohorts. Epilepsy ranged from 30-80% of the children and dysphagia, from 22.2-67.7%. Calcifications and ventriculomegaly were the most consistent and frequent abnormality (around 80%). Cortical atrophy or other development disorders were reported in around 50% of the children. The frequency of fundal, optical nerve and hearing abnormalities ranged from 0-67.1%, 0-36.5% and 0–50%, respectively. This large sample size allowed us to describe the spectrum of severity and of manifestations of Congenital Zika Syndrome.

## Introduction

The Zika virus (ZIKV) epidemic in Brazil, and its consequences for perinatal and childhood health outcomes, have presented lasting challenges for affected families, local health authorities, and the scientific community. In May 2015, ZIKV transmission was confirmed in Brazil [1]. Later that year, an unexpected rise in microcephaly cases among newborns prompted the identification of a link between congenital ZIKV infection and the observed birth defects [2]. Since then, researchers provided evidence showing that the full spectrum of Congenital Zika Syndrome (CZS) extends beyond the initially described characteristics of microcephaly and brain malformations to also include other structural abnormalities and functional deficits [3, 4]. In total 1,828 congenital ZIKV cases have been confirmed in Brazil, with a peak in 2015–2016 followed by a decline in 2017–2018, with sporadic cases in subsequent years, just 4 cases being reported in 2023 [5]. Zika transmission remains in several countries but has generally been at low levels throughout 2018 to the present [6].

Genomic analyses have revealed that the Asian lineage of ZIKV predominated during the Brazilian outbreak [7]. However, data on viral sequences from congenital ZIKV infections remain limited, and the contribution of specific viral mutations to microcephaly pathogenesis remains unclear [8].

Since their initiation in late 2015, individual studies in Brazil have confirmed the link between microcephaly and congenital ZIKV infection and provided evidence showing that the full spectrum of Congenital Zika Syndrome (CZS) extends beyond the initially described characteristics of microcephaly and brain malformations to also include other structural abnormalities and functional deficits [3, 4, 9].

Although the Brazilian cohorts were developed independently at the height of the epidemic, the principal investigators of the studies organized meetings, which were supported by PAHO/WHO, to harmonize protocols and data collection instruments for future pooled analyses [10]. The resultant Zika Brazilian Cohorts-Consortium (ZBC-Consortium) now brings together individual-level data from 12 cohorts of children with CZS and Zika-related microcephaly born between 2015 and 2018 in the North, Northeast and the Southeast regions of Brazil [11]. One of the main gaps in characterizing the CZS up to now is the small number of participants in individual studies. Pooling the data together overcomes the sample size-related limitations of individual studies and provides unique opportunities for investigating heterogeneity between studies and, as appropriate, to better characterize the CZS. This study focuses on the subset of children with Zika-related microcephaly in the ZBC-Consortium and conducts individual-participant data meta-analyses to describe adverse anthropometric, clinical, and neuroimaging outcomes occurring in the first three years of life.

## Methods

### Ethics statement

All participating cohort studies had ethical approval by local ethics committees: Hospital Universitário Oswaldo Cruz (CAAE 52803316.8.0000.5192); Universidade Federal de Pernambuco (CAAE 54734316.5.0000.5208); University of Sergipe (CAAE 53611316.0.0000.5546); Instituto Gonçalo Moniz/Fiocruz-BA (CAAE 51889315.7.0000.0040); Fundação de Medicina Tropical Dr Heitor Vieira Dourado (CAAE 60168216.2.0000.0005); Hospital Universitário do Maranhão (CAAE 65897317.1.0000.5086); Instituto Evandro Chagas (CAAE 56969516.8.0000.0019; CAAE 68067217.0.0000.0019; CAAE 29124920.6.0000.0019); Instituto Fernandes Figueira/Fiocruz-RJ (CAAE 52675616.0.0000.5269); Instituto de Puericultura e Pediatria Martagão Gesteira (CAAE 54497216.2.1001.5264); Hospital das Clínicas da Faculdade de Medicina de Ribeirão Preto da USP (CAAE 56522216.0.0000.5440); and Faculdade de Medicina de Jundiaí (CAAE 53248616.2.0000.5412). All participating pregnant women and persons responsible for participating children provided a written informed consent form.

### Data sources, study population, and inclusion criteria

The ZBC-Consortium identified potentially eligible cohort studies from those that: (i) received funding from one of the major Brazilian research granting institutions; (ii) had participated in the protocol and instrument harmonization meetings held in Recife/Brazil, Mexico City/Mexico, and Geneva/Switzerland; or (iii) were suggested by participating cohorts. All eligible studies, which had or were continuing to follow up children with CZS in Brazil, were invited to participate.

This study included individual participant data from children in the ZBC-Consortium with a definitive, highly probable, or moderately probable diagnosis of CZS and moderate or severe microcephaly; no exclusion criteria were applied.

The diagnosis of CZS was based on the study of França et al. [10], and categorized as (i) ‘definitive’ with laboratory evidence, i.e., immunoglobulin-M enzyme-linked immunosorbent assay (IgM ELISA), plaque reduction neutralization test (PRNT), or quantitative real-time polymerase chain reaction (qRT-PCR) [12] of a ZIKV infection during pregnancy based on maternal or child samples, regardless of other findings; (ii) ‘highly probable’ with brain imaging features suggestive of ZIKV infection (i.e., calcifications, cerebral atrophy, ventriculomegaly, cortical development disorder, cerebellum hypoplasia/atrophy, brainstem hypoplasia/atrophy and agenesis/dysgenesis of the corpus callosum [4]) and negative serological results for syphilis, toxoplasmosis and cytomegalovirus; and (iii) ‘moderately probable’ with brain imaging features suggestive of ZIKV infection but without results or with inconclusive results for one or more of the three alternative congenital infections (i.e., syphilis, toxoplasmosis and cytomegalovirus).

Microcephaly was defined as (i) moderate with a head circumference (HC) z-score of>-3 SD to ≤-2SD below the mean or (ii) severe with a HC z-score of ≤-3SD below the mean. To assure standardization across studies, we recalculated head circumference z-scores for all children in the ZBC-Consortium using the following approach. When data were available from the time of birth, we calculated the sex and gestational age-specific HC z-score for both preterm and term newborns using the INTERGROWTH-21st curves [13]. When the first measurement of HC was performed after birth for preterm children, we calculated the sex and gestational age-specific HC z-score using INTERGROWTH-21st curves until the completion of 64 weeks of gestational age and, thereafter, using the sex-specific WHO-Anthro curves [14]. Microcephaly cases were further classified as disproportionate based on the relationship of HC to length [15].

### Data abstraction and categorization

Due to protocol harmonization initiatives, most cohorts in the ZBC-Consortium have collected data in a similar way. The studies included children born from 2015 onwards and the latest evaluation was on October 17, 2019. Following comparisons between questionnaires from individual studies, a common questionnaire with a standardized codebook was developed and distributed to participating studies. Participating studies securely transferred the individual participant data relevant to the common questionnaire to a data center established at the Oswaldo Cruz Foundation’s (Fiocruz-Recife, Pernambuco/Brazil). Data for the ZBC-Consortium were securely stored on a single platform built using GeneXus-X development and aspx code.

In the current study we evaluated: severity of microcephaly, proportionality, prematurity (gestational age of <37 weeks at delivery), low birth weight (birth weight of <2500g at delivery), small for gestational age (z-score for birth weight of <-1.28 at delivery for sex and gestational age), large for gestational age (z-score for birth weight of >1.28 at delivery for sex and gestational age).

Congenital malformations included: congenital clubfoot, distal arthrogryposis, generalized arthrogryposis, umbilical hernia, inguinal hernia, congenital hip dislocation, prominent occiput, girata skin, overlapping cranial bones (palpable sutures), excess frontal skin, excess skin on the neck, excess skin on the back, epicantus, strabismus, nystagmus, microphthalmos, eyelid ptosis, absence of lingual frenulum, retrognathia, nevus in the face.

Neurological disorders were grouped and categorized as: social attention deficits (e.g., lack of a responsive social smile, referred irritability, does not fixe gaze and does not follow with eyes, or lack of auditory or visual response), persistence of primitive reflexes (Moro, asymmetrical tonic neck reflex, palmar grasp, plantar grasp, gait, Babkin, and Galant reflexes) [16], spastic hypertonia (at >3 months of age), osteotendinous hyperreflexia (bicipital, triceps, radial, patellar, and Achilles tendons), abnormalities of the Landau reflex (extensor tonus of the neck and trunk), cranial nerve syndrome [grouped into three classes: Group 1 (deficit in II, III, IV, or VI cranial nerves), Group 2 (deficits in VII and VIII cranial nerves), and Group 3 (deficits in X, XI, or XII cranial nerves)], motor deficit syndrome (motor underactivity, abnormal movements, abnormal ocular motricity), and epilepsy (seizure identified by caregiver report, home video, or attending physician observation and/or EEG abnormalities [17–19]. Neuroimaging abnormalities included the overall abnormal imaging results based on ultrasound (US), computerized tomography (CT), or magnetic resonance imaging (MRI) as well as the specific outcomes of calcifications, ventriculomegaly, agenesis/dysgenesis of the corpus callosum, and hypoplasia/atrophy of the cerebellum.

Structural ocular abnormalities, detected either by fundoscopy or RetCam, included fundal, optical nerve, and retinal abnormalities. For those sites (Rio de Janeiro-Universidade Federal do Rio de Janeiro-UFRJ; Recife-Microcephaly Epidemic Research Group-MERG; São Luis) which specified the retinal abnormalities (104 children), the findings described were focal pigment mottling, chorioretinal atrophy/scars and vascular abnormalities. As some sites did not detail the type of structural ocular abnormality but informed that there was an abnormality, the term fundal abnormality included this group in addition to the others in which the type of abnormality was described. Audiologic abnormalities (by auditory brainstem response (ABR) test), and dysphagia (based on caregiver report and/or clinical assessment by specialists).

### Statistical analysis

In the analysis we used a logistic-normal model with the command Metaprop_one in Stata to explore heterogeneity and estimate the study specific proportions with 95% Wilson score confidence intervals, the pooled estimate and respective confidence interval and the tau-squared (*τ*^2^), on the logit scale [20]. We also conducted a sensitivity analysis, shown in the supplement, using the Freeman-Tukey double arcsine transformation with the command Metaprop, estimating the same parameters plus the I-squared (I^2^) statistics. The logistic-normal model does model the true binomial distribution of the data and therefore, it is good for all proportions. The Freeman-Tukey is designed to stabilize the variance of proportions, especially when handling small sample sizes orextreme proportions near 0 or 1, which occurred for some variables in our study. The I^2^ is a relative measure, expressing the percentage of the total variation in study estimates that is due to heterogeneity, and *τ*^2^ is an absolute measure of heterogeneity which represents the between-study variance. *τ*^2^ provides information of how the effectsize varies across populations. Using Metaprop_one, we also calculated the Chi-squared statistic which is related to *τ*^2^; the Likelihood Ratio test: Random effect vs Fixed effect Model Chi-squared (LR test: RE vs FE Model chi^2) tests the null hypothesis that *τ* ^2^ = 0, i.e., that there is no between-study variance, meaning that there is no heterogeneity. With Metaprop, the Chi-squared calculated tests the null hypothesis that all studies have a common effect size using the Cochran’s Q statistic. Descriptive analysis was performed using mean and standard deviations for continuous variables with normal distributions and using median and interquartile ranges for variables without normal distributions. Categorical data were presented by absolute and relative frequencies. All analyses were performed using STATA, version 14 (College Station, TX, USA).

### Data availability policy

Data generated and analyzed during this study is included in the article and its supplementary information files. As our underlying data could be stratified geographically and includes sensitive health information and other personal characteristics, there is a risk that individual children and their families could be identified if the data were shared publicly. While we cannot share the data publicly, de-identified data can be made available upon reasonable request from qualified investigators by contacting the Programa de Pós-Graduação em Ciências da Saúde (PPGCS) da Universidade de Pernambuco (UPE) at ppg.cienciasdasaude@upe.br.

## Results

We included 843 children, of which 542 (64.3%) with definitive CZS diagnoses, 127 (15.1%) highly probable, and 35 (4.1%) moderately probable, based on França et al. criteria. It was not possible to classify 139/843 (16.5%) children with microcephaly according to these criteria. Microcephaly was diagnosed at birth in 601 of the 843 (71.3%) children. A total of 58 of the 843 (6.9%) children were diagnosed with microcephaly at first evaluation as there was no measure of HC at birth; the median age of evaluation was 31.6 months (IQR 0.4-40.1). From the 843 children, 172 (20.4%) that had no microcephaly at birth, developed microcephaly over follow-up, i.e., postnatal microcephaly. For the remaining 12 of the 843 (1.4%) children there was information on the presence of microcephaly but the HC measure was not provided. In the group of children with microcephaly diagnosed at birth 217 (36.1%) were classified as having moderate and 384 (63.9%) as having severe microcephaly. In those diagnosed at first evaluation, 8 (13.8%) had moderate and 50 (86.3%) had severe microcephaly. In the group with postnatal microcephaly, 63 (36.6%) had moderate and 109 (63.4%) had severe microcephaly. Microcephaly was not classified according the severity for the 12 children for which the HC measure was not informed.

**Fig 1:**
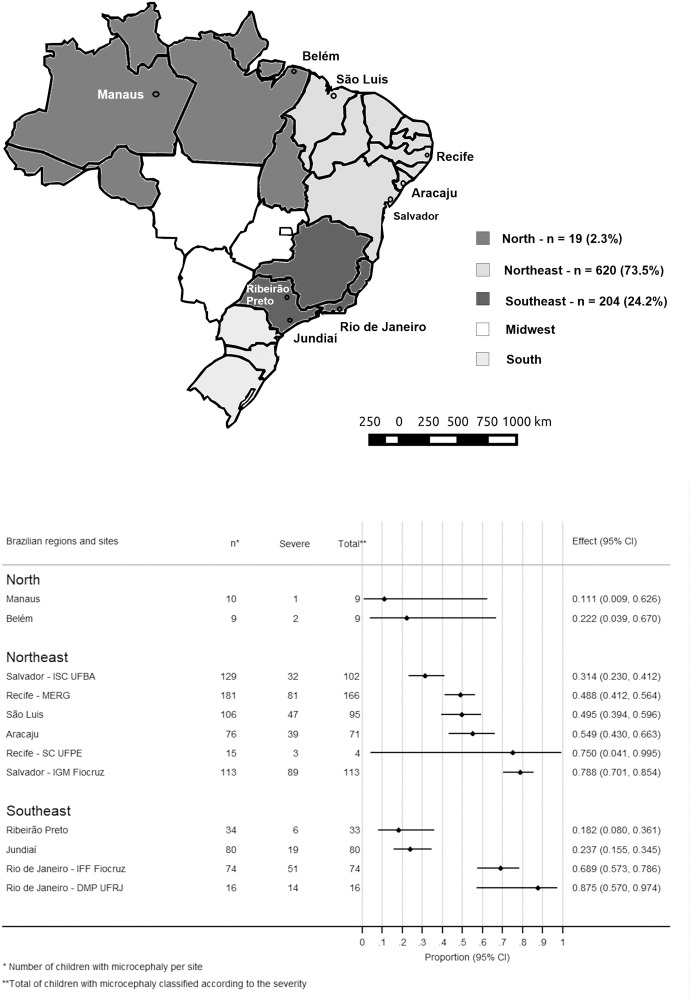
Participating cohorts of the ZBC-Consortium and severity of microcephaly per site. A. Distribution of the cohorts participating in the ZBC-Consortium by Brazilian Regions. Note: Link to the base layer of the map: https://pixabay.com/pt/vectors/brasil-geografia-mapa-estados-153881/; Link of the terms of use/license information for the base layer of the map: https://pixabay.com/pt/service/license-summary/. B. Frequency of severe microcephaly in the ZBC-Consortium per site, city, and Brazilian Region.

The study participants originated from cohorts in three out of the five Brazilian Regions. The North Region comprised 2.3% of the total sample, with 19 participants, being 9 from Belém and 10 from Manaus. The Northeast Region comprised 73.5% of the total sample, with 620 participants, being 76 from Aracaju; 181 from MERG [21] and 15 from Saúde Coletiva-Universidade Federal de Pernambuco both in Recife; 106 from São Luis; 129 from Instituto de Saúde Coletiva-Universidade Federal da Bahia and 113 from Instituto Gonçalo Moniz-Fiocruz both in Salvador. The Southeast Region comprised 24.2% of the total sample, with 204 participants, being 80 from Jundiaí [22]; 34 from Ribeirão Preto; 74 from Instituto Fernandes Figueira-Fiocruz and 16 from UFRJ both in Rio de Janeiro (Fig 1).

The p-value of the LR test (RE vs FE Model) for the frequency of severe microcephaly was 0,000 suggesting heterogeneity between sites. The pooled estimate of the frequency of severe microcephaly was 47.1% across study sites, ranging from 11.1 to 87.5% (Fig 1B).

The frequency of disproportionate microcephaly (i.e., classified in n = 601 children evaluated at birth) ranged from 15.8 to 93.3% overall, being 10.4 to 88.9% in children with moderate microcephaly and 64.2 to 92.8% in children with severe microcephaly. (Overall:LR test: RE vs FE Model p = 0.000, τ^2 ^= 1.376**)**; Moderate: LR test: RE vs FE Model p = 0.000, τ^2 ^= 1.211; Severe: LR test: RE vs FE Model p = 0.000, τ^2 ^= 1.130). The site Recife-SC UFPE was excluded of the analysis for disproportionate microcephaly overall and moderate microcephaly because of the low number of children with severe microcephaly. For the analysis of disproportionate microcephaly in children with severe microcephaly the sites Manaus, Belém, Recife-SC UFPE and Ribeirão Preto were excluded because of the low number.

Although, there was heterogeneity between sites in relation to the occurrence of microcephaly at birth, severity of microcephaly, and abnormal CNS image exam, the overall pooled estimated frequency of these variables was higher in children with disproportionate than in those with proportionate microcephaly. The overall pooled estimated frequency of microcephaly at birth in children with disproportionate and proportionate microcephaly was respectively 95.7% (95% CI 84.2–98.9) and 63.6% (95% CI 42.1–80.8); of severe microcephaly in children with disproportionate and proportionate microcephaly was 66.6% (95% CI 51.8–78.7) and 25.0% (95% CI 16.9-35.3 respectively. The frequency of neurological disorders in children with disproportionate and proportionate microcephaly was 72.8% (95% CI 59.6–82.9) and 64.2% (95% CI 57.6–70.3), respectively.

Among the 680 microcephalic children with available data on congenital malformations (Table 1), more than one-fifth presented with: epicantus (40.1%), prominent occiput (39.2%), excess skin on the neck (26.7%), overlapping cranial bones (24.2%), strabismus (23.7%), retrognathia (23.5%), girata skin (22.4%).

**Table 1:** Congenital malformations in children with Zika-related microcephaly participating in the ZBC-Consortium.

Malformation	Microcephaly at birth or later (n = 680 congenital malformations of children with microcephaly)			Lower frequency	Higher frequency	τ^2^	RE *versus* FE model
	Case/total*	%**	95% CI**				
Congenital clubfoot	44/322	8.8%	4.0 – 18.4	0%	28.6%	0.528	0.001
Distal arthrogryposis	64/295	15.4%	6.4 – 32.9	0%	31.1%	0.595	0.001
Generalized arthrogryposis	30/304	3.6%	0.0 – 20.8	0%	15.1%	1.446	0.004
Umbilical hernia	36/310	5.0%	0.0 – 38.8	0%	100%	5.225	0.006
Inguinal hernia	15/319	1.8%	0.0 – 20.6	0%	76.9%	8.521	0.001
Congenital hip dislocation	37/305	6.5%	2.3 – 17.2	0%	18.7%	0.584	0.002
Prominent occiput	107/273	39.2%	33.6 – 45.1	35.9%	44.9%	0.000	1.000
Girata skin	55/270	22.4%	8.2 – 48.3	0%	71.4%	1.060	0.320
Overlapping cranial bones (palpable sutures)	67/283	(24.2%	18.2 – 31.4	14.3%	42.9%	0.032	0.274
Excess frontal skin	31/182	17.0%	12.2 – 23.2	0%	18.2%	0.000	1.000
Excess skin on the neck	72/270	26.7%	21.7 – 32.3	22.7%	71.4%	0.000	1.000
Excess skin on the back	22/182	12.1%	8.1 – 17.7	0%	30.0%	0.000	1.000
Epicantus	73/182	40.1%	33.2 – 47.4	0%	50.0%	0.000	1.000
Strabismus	106/334	23.7%	12.2 – 40.9	7.9%	44.3%	0.581	0.001
Nystagmus	69/350	13.1%	4.9 – 30.6	0%	37.2%	0.745	0.001
Microphthalmos	13/253	3.5%	0 – 13.5	0%	7.4%	0.395	0.183
Eyelid ptosis	12/181	6.6%	0.4 – 11.3	0%	7.3%	0.000	1.000
Absence of lingual frenulum	29/286	2.4%	0 – 21.1	0%	17.2%	2.510	0.001
Retrognathia	43/183	23.5%	17.9 – 30.2	0%	30.0%	0.000	1.000
Nevus in the face	9/228	2.4%	0 – 34.0	0%	5.5%	0.496	0.354

* Differences in denominators are due to missing values

** Random pooled estimative

**Fig 2:**
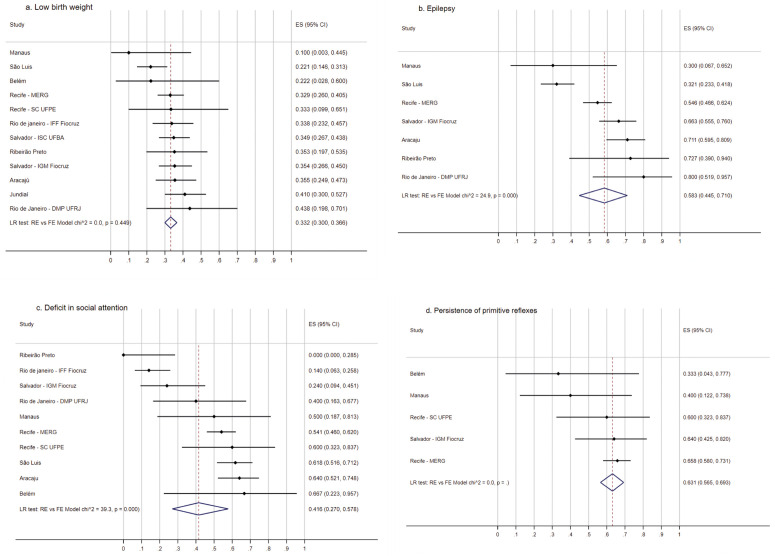
Frequency of low birth weight and neurological abnormalities of children with Zika-related microcephaly of the ZBC-Consortium. Frequency of low birth weight (A), epilepsy (B), deficit of social attention (C) and persistence of primitive reflexes (D) in children with Zika-related microcephaly of the ZBC-Consortium.

The frequency of low birth weight was 33.2% varying from 10 to 43.8%, but for most of the sites it was between 20 and 40% (LR test: RE vs FE Model p = 0.449) (Fig 2A). For small for gestational age and large for gestational age there was also no heterogeneity (S1B Fig and S1C), while prematurity, and dysphagia presented with heterogeneity and a high variability through sites (S1A Fig and S1D).

The neurological manifestations among the children considered each site reported. Several neurological findings, including those that are proxy of severe injury were very frequent through studies sites; deficit of social attention (lack of social interactions) was reported in at least 50% of the children in the cohort studies with higher number of participants (Fig 2C). The same was observed on spastic hypertonia (S2B Fig). Epilepsy was reported in 30–80% (LR test: RE vs FE Model p = 0.000, overall pooled estimate = 58.3, 95% CI 44.5-71.0) (Fig 2B). Persistence of primitive reflexes (Fig 2D) was investigated in five sites and there was homogeneity between sites with an overall pooled estimate of 63.1 (CI 56.5-69.3).

**Fig 3:**
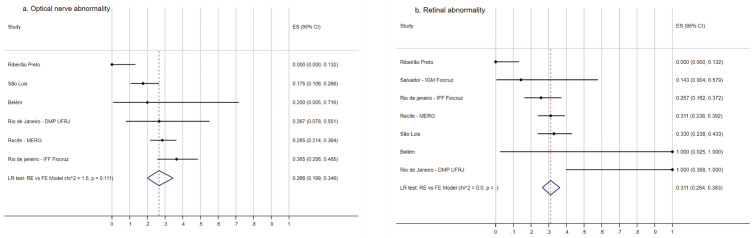
Frequency of ocular abnormalities in children with Zika-related microcephaly participating in the ZBC-Consortium. Frequency of optical nerve (A) and retinal (B) abnormalities in children with Zika-related microcephaly participating in the ZBC-Consortium.

The frequency of eye fundal abnormality among the children ranged from 0 to 67.1% (LR test: RE vs FE Model p = 0.000, overall pooled estimate = 49.8, 95% CI 39.0-60.7) (S2A Fig) and the frequency of optical nerve abnormality varied from 0 to 36.5% (LR test: RE vs FE Model p = 0.111, overall pooled estimate = 26.6, 95% CI 19.9-34.6) (Fig 3A), and the frequency of retinal abnormality varied from 0 to 100% (LR test: RE vs FE Model p = 1.000, overall pooled estimate = 31.1, 95% CI 26.4-36.3) (Fig 3B).

**Fig 4:**
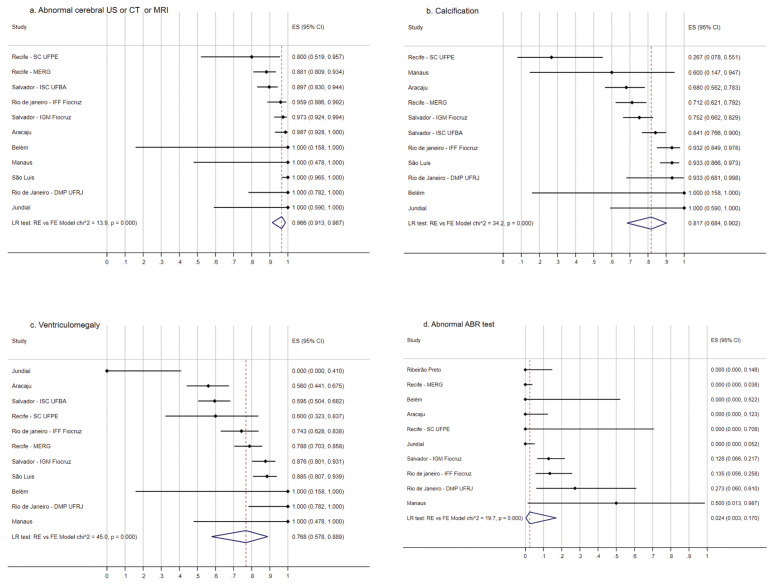
Frequency of imaging and audiologic abnormalities in children with Zika-related microcephaly participating in the ZBC-Consortium. Frequency of abnormal cerebral ultrasonography, or tomography, or magnetic resonance imaging (A), calcification (B), ventriculomegaly (C) and abnormal ABR test (D) in children with Zika-related microcephaly participating in the ZBC-Consortium.

In Fig 4A we observe that for five sites, all studied children had at least one abnormal CNS image exam, and for the other six, at least 80%. Calcifications and ventriculomegaly were the most consistent and frequent described abnormality, calcification ranging from 26.7 to 100% (LR test: RE vs FE Model p = 0.000, overall pooled estimate = 81.7, 95% CI 68.4-90.2) (Fig 4B) and for ventriculomegaly, the frequency was above 55% for 10 of the 11 sites (LR test: RE vs FE Model p = 0.000, overall pooled estimate = 76.8, 95% CI 57.8-88.9) (Fig 4C). Spastic hypertonia was found in seven sites in which they were investigated, with a frequency of 100% in four sites (S2B Fig). For cerebellum hypoplasia/atrophy and agenesis/dysgenesis of the corpus callosum there was heterogeneity, and the frequency of cerebellum hypoplasia/atrophy varied from 0 to 50% and of agenesis/dysgenesis of the corpus callosum, varied from 0 to 100% (S2C Fig and S2D Fig).

Although an audiology exam was not performed in all the cohort studies, abnormalities were not as common as CNS or eye disease. The frequency of abnormal ABR test was 0% in six out of 10 sites (Fig 4D).

The forest plot graphics with a sensitive analysis, using the Freeman-Tukey double arcsine transformation, estimating the same parameters plus the I-squared (I^2^) statistics, are shown in supplement (S3 Fig to S7 Fig). The results of the pooled estimates were very similar in both approaches and for most of the variables the I^2^ was above 75%, suggesting high heterogeneity.

## Discussion

This study brings together individual participant data from 12 prospectively followed pediatric cohorts from three regions of Brazil that were started during the ZIKV epidemic. Although the frequencies of individual abnormalities were generally heterogeneous across the studies, most children with Zika-related microcephaly presented with a very similar spectrum of signs and symptoms, regardless of geographic origin, with the most common abnormalities related to neurologic function, ocular structure, and hearing. Similar to our findings from Brazil, previous research from the French territories in the Americas, the United States territories, and Colombia reported neurological, ophthalmological, and hearing abnormalities as the most frequent outcomes in children with prenatal ZIKV exposure [23, 24].

We observed a notable overlap between microcephaly and other congenital malformations, with the most frequent being epicantus, prominent occiput, excess skin on the neck and overlapping cranial bones. Prominent occiput and excess skin on the neck are due to the skull deformity associated with the severity of microcephaly. The other malformations appear to be part of the spectrum of manifestations of CZS.

We also observed a high frequency of children who were born with low birth weight (20–40%) and/or small for gestational age (30–65%). These findings align with a large body of evidence suggesting that congenital infection from pathogens, such as *Toxoplasma gondii*, rubella virus, cytomegalovirus (CMV), herpes simplex virus (HSV), and *Treponema pallidum* (syphilis), may result in intrauterine growth restriction (IUGR) [25]. Strikingly, a high number of children (n = 394) in this study presented with disproportionate microcephaly. As the frequency of severe microcephaly and abnormal CNS image exam was higher among children with disproportionate microcephaly compared to those proportionate microcephaly, we hypothesize that brain damage may have been the main cause of the disproportion.

A range of neurological signs/symptoms were also very frequent in children with Zika-related microcephaly. In the pooled analyses, we observed high frequencies of social attention deficits, the persistence of primitive reflexes, and epilepsy. The occurrence of neurologic abnormalities likely represents both direct (e.g., ZIKV’s tropism for neural progenitor stem cells) and indirect (e.g., inflammatory responses that compromise neurogenesis) consequences of the presence of ZIKV in the CNS during fetal development [26]. Further research is warranted to better understand the underlying mechanisms and how factors associated with vertical transmission, such as the timing of infection and/or the viral load, may influence the frequency of ZIKV-related neurologic abnormalities. Further investigation is also necessary to clarify if attention deficits in these children is due to development delay and/or autism and/or if they are sensory impaired with low vision or hearing loss.

Consistent with the high frequency of neurologic abnormalities, postnatal brain assessments based on US, CT, and/or MRI imaging found abnormalities in several sectors of the brain. In the pooled analyses, we observed high frequencies of calcifications, ventriculomegaly, and cerebral atrophy. These results are broadly consistent with MRI-based animal model studies, which showed that ZIKV-infected infant rhesus macaques had ventriculomegaly, hypotrophy/atrophy, and altered functional connectivity between brain areas (e.g., amygdala, hippocampus, cerebellum). Further, the neuropathological alterations corresponded with neuroimaging results and were consistent with the behavioral and memory deficits [27]. This type of finding is consistent with that we observed in the children exposed to ZIKV [28].

Ocular structural abnormalities were similar to those reported by other authors in Brazil and elsewhere, mainly optic nerve pallor/hypoplasia, focal pigment mottling and chorioretinal scars [29]. The hearing evaluations we showed were based just on ABR test. More detailed by Cortical Auditory Evoked Potentials in a small number of children suggested that regardless of changes in the CNS, this population appears to have, to some extent, the cortical ability to process sound stimuli, however, the presence of microcephaly may interfere with the attention capacity and auditory discrimination [30].

In the pooled analyses, dysphagia was common and observed in 46.9% of children. The substantial heterogeneity is likely to be due to differences in case ascertainment approaches across the study sites.

The findings described in this study were those that were investigated in most of the sites, as they were part of the manifestations of the CZS initially described. However, the broader description of the CZS spectrum must be complemented by the findings of specific studies that used more sophisticated instruments to investigate some abnormalities, such as manifestations of the urogenital system [31], endocrinological [32], cardiorespiratory [33, 34] and gastrointestinal [35]. Additional and equally important information should come from studies evaluating the neurodevelopment of these children as they reach different age groups [36].

Although there was heterogeneity for most of the variables, it does not seem that there is likely to be a biological difference (e.g., in relation to underlying comorbidities or consequences of congenital infection) between the children in the different cohorts within the ZBC-Consortium. There are several possible study design-related explanations for the heterogeneity in the frequency of some characteristics across studies. First, each study used different recruitment criteria (e.g., some recruited antenatally, such as from pregnancy cohorts, while others recruited from outpatient pediatric clinics, primary care settings, or from the Municipal Health Surveillance database which were subsequently contacted by the local primary care teams). These different recruitment criteria could introduce selection biases (e.g., sites recruiting as part of surveillance efforts may have had a higher sensitivity for recruiting children with milder CZS). Of note, the frequency of severe microcephaly varied from 11 to 87.5% across cohorts. We believe that this variation is probably related to different inclusion criteria among sites, as some sites primarily recruited children with phenotypes more typical for severe rather than moderate microcephaly. Second, since data were collected from studies conducted concurrently, without a previous standardization, and retrospectively harmonized, outcome definitions may have differed to some extent. Notably, each study relied on the expertise of local evaluators, and potentially differing techniques and instruments, to diagnose the abnormalities in the context of a new congenital syndrome never before described. In addition to differences between studies, the learning curve over the duration of follow-up within a given site must be considered.

An important strength of this study was the large sample size, pooling the individual primary data of 843 children with Zika-related microcephaly, a large proportion of the 1,828 confirmed CZS cases notified between 2015 and 2023 in Brazil [5]. This study has some limitations: i) children were enrolled since the start of the epidemic, and therefore the laboratory confirmation was not possible for all of them. This is the reason why we worked with different levels of evidence of ZIKV infection, the criteria adopted by Brazilian researchers [10]; ii) there may have been some degree of misclassification of CZS, but it is not likely that it may have affected substantially our results as only 4.1% of the children had a level of evidence of moderately probable. In relation to the fact that children with microcephaly born to mothers who presented ZIKV infection during pregnancy had the diagnosis of CZS categorized as ‘definitive’, it may not be excluded that they had microcephaly of another aetiology, but if it occurred was probably in a very small proportion as microcephaly is a rare event and again it is not likely that it would bias our results.

In conclusion, this study presents the findings from the largest ever analysis of children with Zika-related microcephaly. This relatively large sample size allowed us to observe that, among children with microcephaly, there exists a spectrum in the severity and types of manifestations of CZS. Despite the heterogeneity found across the different sites, structural abnormalities of the central nervous system, detected by neuroimaging, and abnormalities in neurological and ophthalmological examination were the most frequently reported outcomes. This severe CNS damage demands multidisciplinary care and assistance from different medical and other health specialties.

## Supporting information

S1 Fig Frequency of prematurity, small for gestational age and large for gestational age and dysphagia of children with Zika-related microcephaly of the ZBC-Consortium. Frequency of prematurity (A), small for gestational age (B), large for gestational age (C), dysphagia (D) in children with Zika-related microcephaly of the ZBC-Consortium.
(TIF)

S2 Fig Frequency of fundal abnormality among the children, spastic hypertonia, cerebellum hypoplasia/atrophy and agenesis/dysgenesis of the corpus callosum of children with Zika-related microcephaly of the ZBC-Consortium. Frequency of fundal abnormality among the children (A) spastic hypertonia (B), cerebellum hypoplasia/atrophy (C) and agenesis/dysgenesis of the corpus callosum (D) in children with Zika-related microcephaly of the ZBC-Consortium.
(TIF)

S3 Fig Frequency of low birth weight and neurological abnormalities of children with Zika-related microcephaly of the ZBC-Consortium (using Freeman-Tukey Double Arcsine Transformation). Frequency of low birth weight (A), epilepsy (B), deficit of social attention (C) and persistence of primitive reflexes (D) in children with Zika-related microcephaly of the ZBC-Consortium.
(TIF)

S4 Fig Frequency of ocular abnormalities in children with Zika-related microcephaly participating in the ZBC-Consortium (using Freeman-Tukey Double Arcsine Transformation). Frequency of optical nerve (A) and retinal (B) abnormalities in children with Zika-related microcephaly participating in the ZBC-Consortium.
(TIF)

S5 Fig Frequency of imaging and audiologic abnormalities in children with Zika-related microcephaly participating in the ZBC-Consortium (using Freeman-Tukey Double Arcsine Transformation). Frequency of abnormal cerebral ultrasonography, or tomography, or magnetic resonance imaging (A), calcification (B), ventriculomegaly (C) and abnormal ABR test (D) in children with Zika-related microcephaly participating in the ZBC-Consortium.
(TIF)

S6 Fig Frequency of prematurity, small for gestational age and large for gestational age and dysphagia of children with Zika-related microcephaly of the ZBC-Consortium (using Freeman-Tukey Double Arcsine Transformation). Frequency of prematurity (A), small for gestational age (B), large for gestational age (C), dysphagia (D) in children with Zika-related microcephaly of the ZBC-Consortium.
(TIF)

S7 Fig Frequency of fundal abnormality among the children, spastic hypertonia, cerebellum hypoplasia/atrophy and agenesis/dysgenesis of the corpus callosum of children with Zika-related microcephaly of the ZBC-Consortium (using Freeman-Tukey Double Arcsine Transformation). Frequency of fundal abnormality among the children (A) spastic hypertonia (B), cerebellum hypoplasia/atrophy (C) and agenesis/dysgenesis of the corpus callosum (D) in children with Zika-related microcephaly of the ZBC-Consortium.
(TIF)
