# Supplementary figures and images for: Characterization of 843 children with Zika-related microcephaly in the first three years of life: An individual participant data meta-analysis of 12 cohorts in the Zika Brazilian Cohorts consortium
Source: PLOS Glob Public Health. 2025 Dec 29;5(12):e0005425. doi: 10.1371/journal.pgph.0005425 (PMC12747325; doi:10.1371/journal.pgph.0005425)

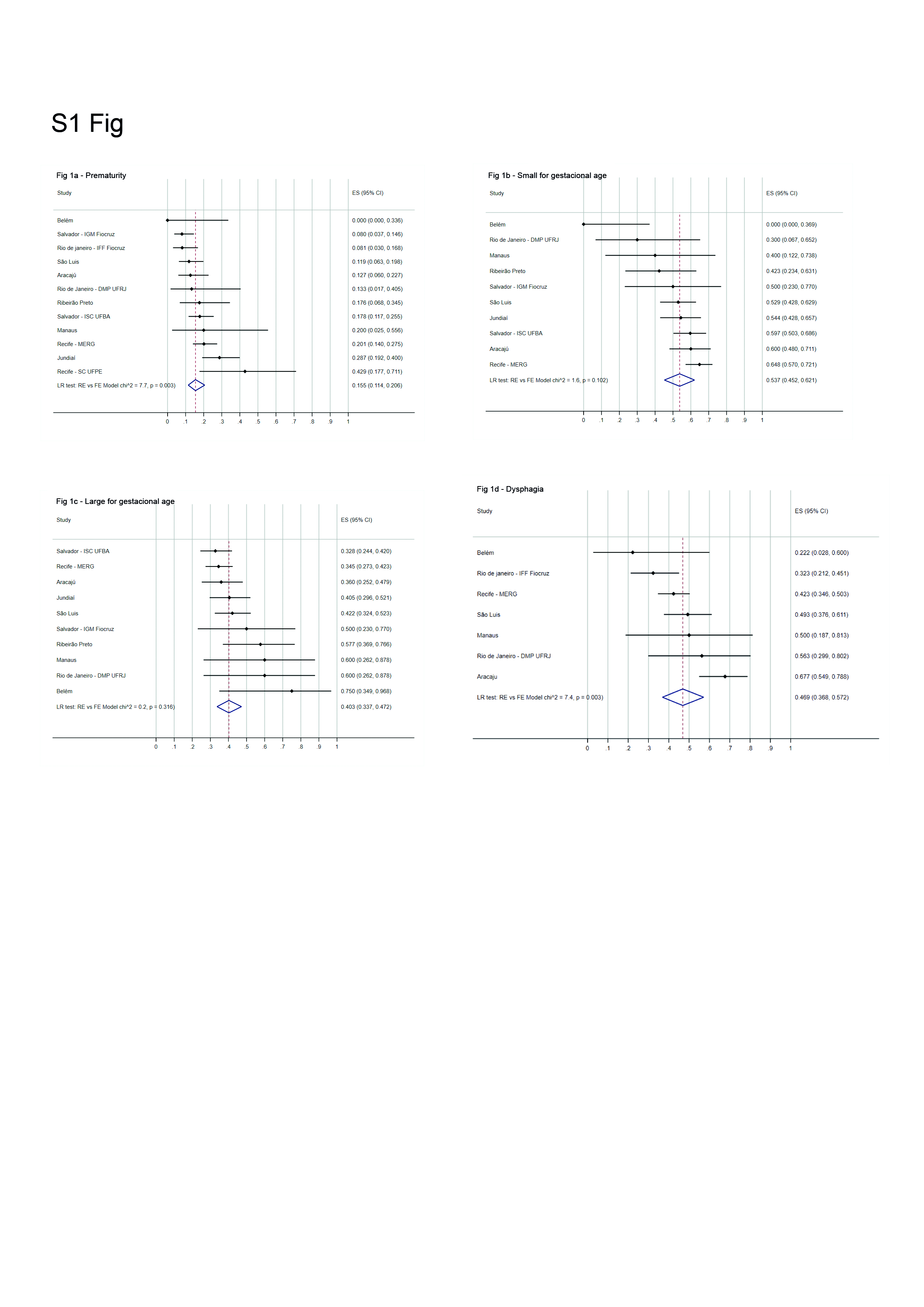

Supplement: S1 Fig — Frequency of prematurity (A), small for gestational age (B), large for gestational age (C), dysphagia (D) in children with Zika-related microcephaly of the ZBC-Consortium. (TIF) [file pgph.0005425.s001.tif]

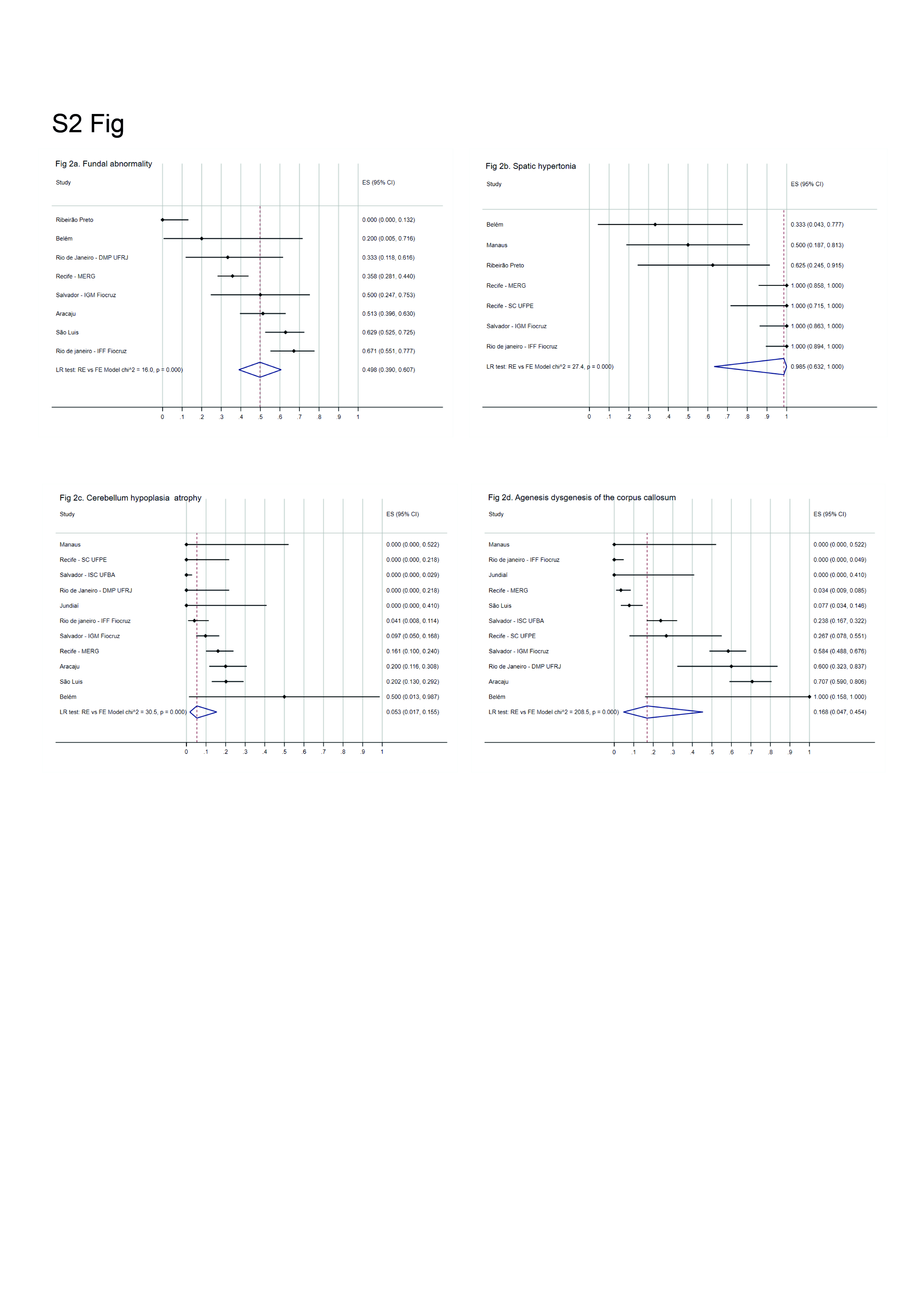

Supplement: S2 Fig — Frequency of fundal abnormality among the children (A) spastic hypertonia (B), cerebellum hypoplasia/atrophy (C) and agenesis/dysgenesis of the corpus callosum (D) in children with Zika-related microcephaly of the ZBC-Consortium. (TIF) [file pgph.0005425.s002.tif]

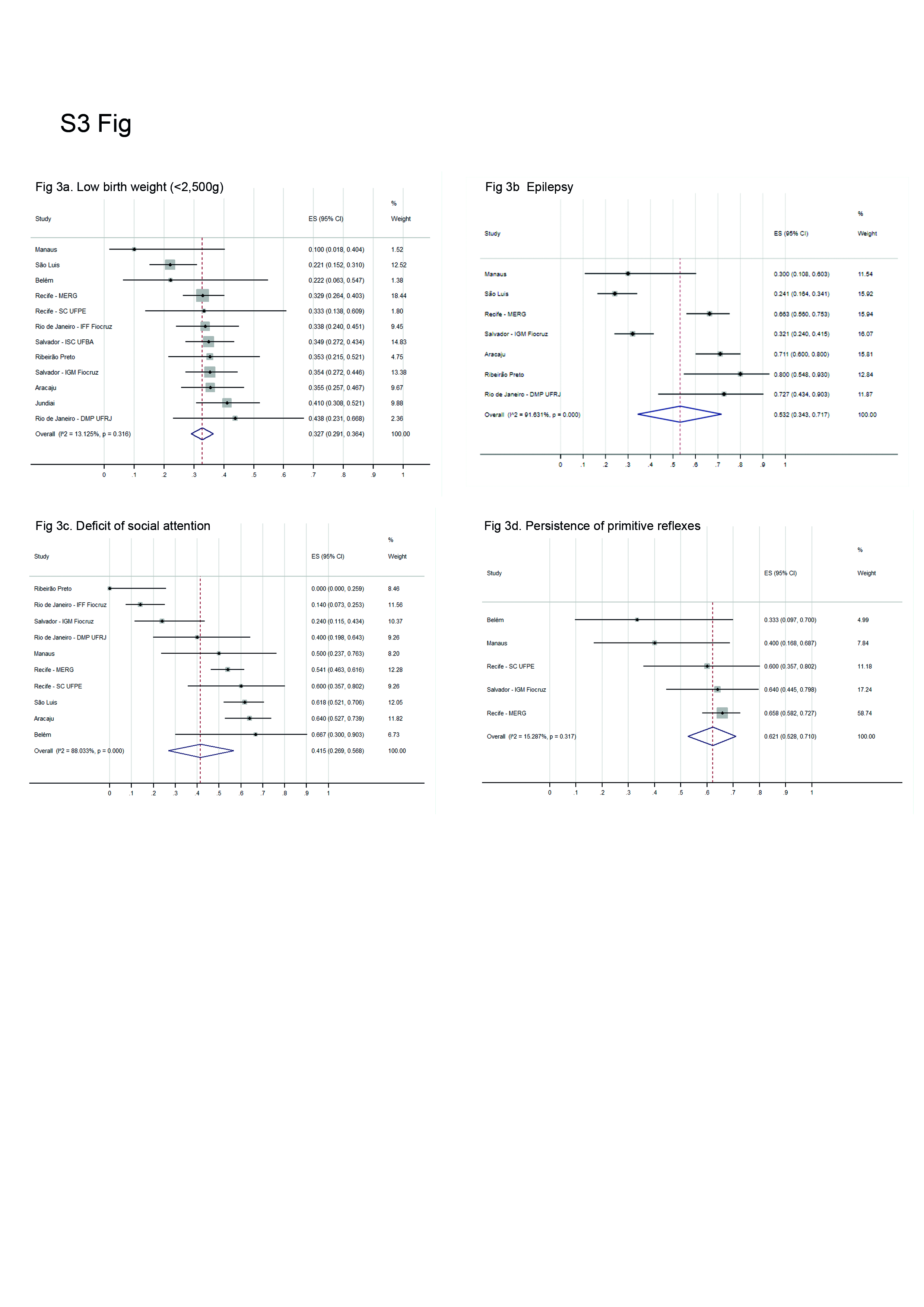

Supplement: S3 Fig — Frequency of low birth weight (A), epilepsy (B), deficit of social attention (C) and persistence of primitive reflexes (D) in children with Zika-related microcephaly of the ZBC-Consortium. (TIF) [file pgph.0005425.s003.tif]

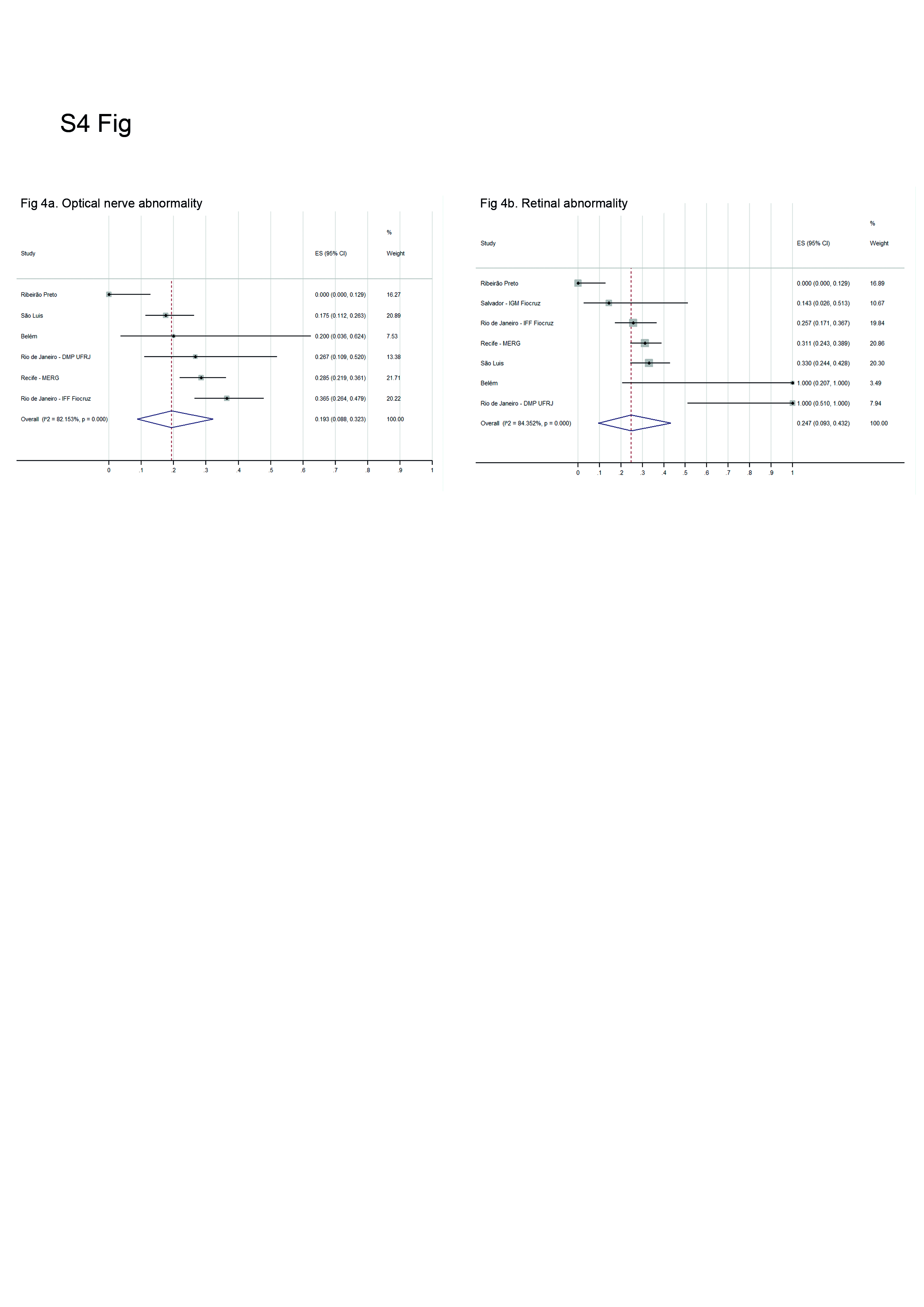

Supplement: S4 Fig — Frequency of optical nerve (A) and retinal (B) abnormalities in children with Zika-related microcephaly participating in the ZBC-Consortium. (TIF) [file pgph.0005425.s004.tif]

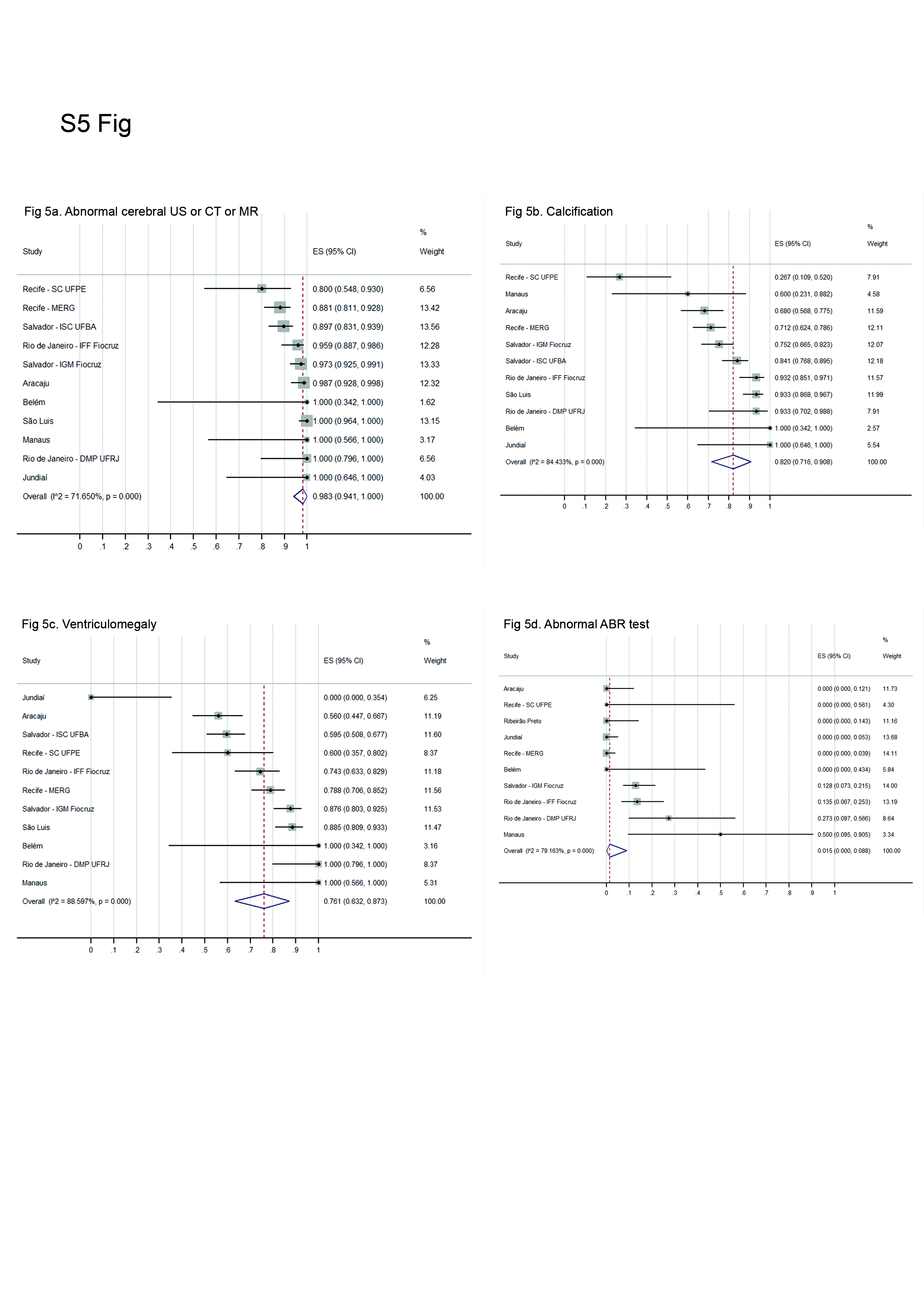

Supplement: S5 Fig — Frequency of abnormal cerebral ultrasonography, or tomography, or magnetic resonance imaging (A), calcification (B), ventriculomegaly (C) and abnormal ABR test (D) in children with Zika-related microcephaly participating in the ZBC-Consortium. (TIF) [file pgph.0005425.s005.tif]

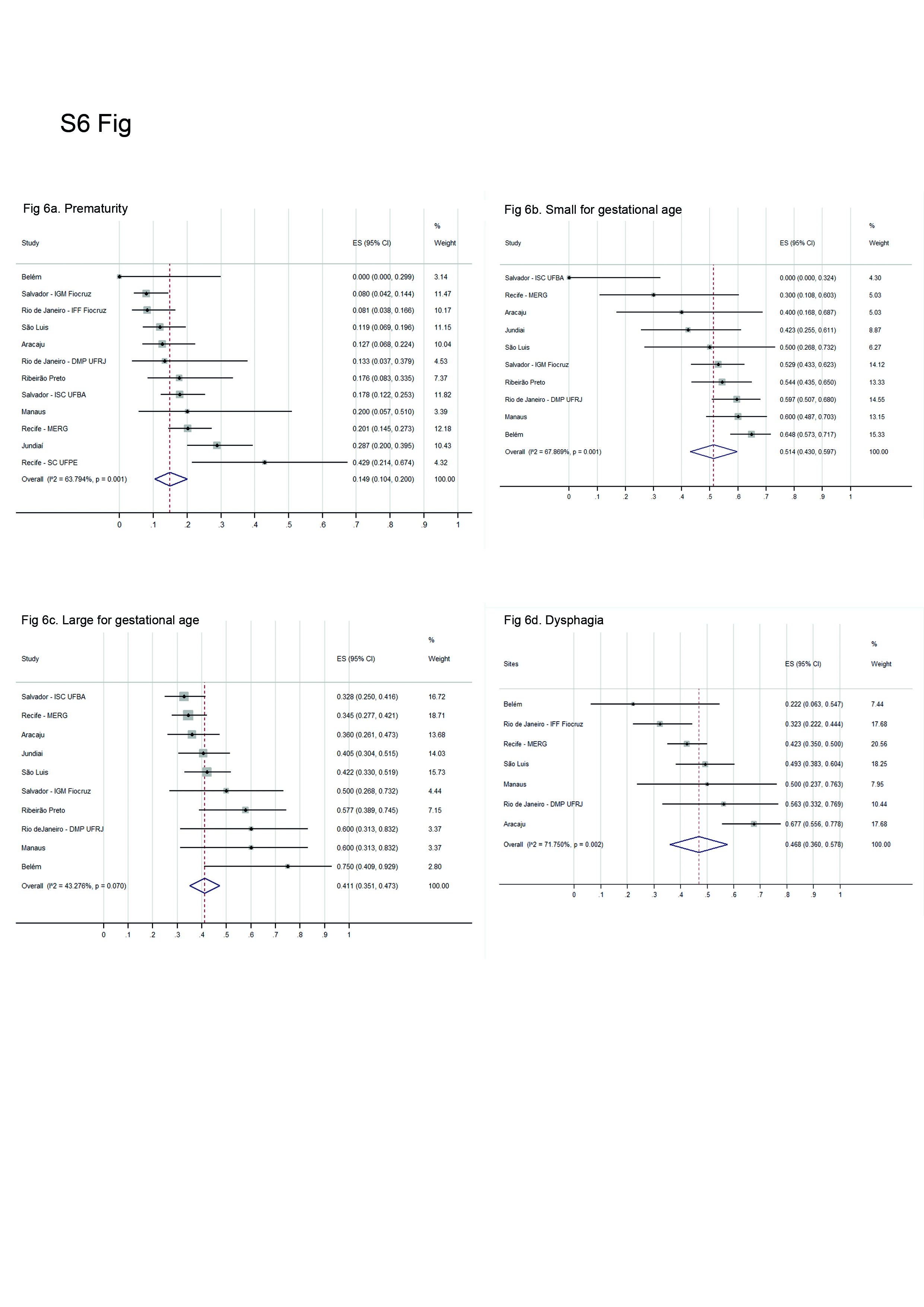

Supplement: S6 Fig — Frequency of prematurity (A), small for gestational age (B), large for gestational age (C), dysphagia (D) in children with Zika-related microcephaly of the ZBC-Consortium. (TIF) [file pgph.0005425.s006.tif]

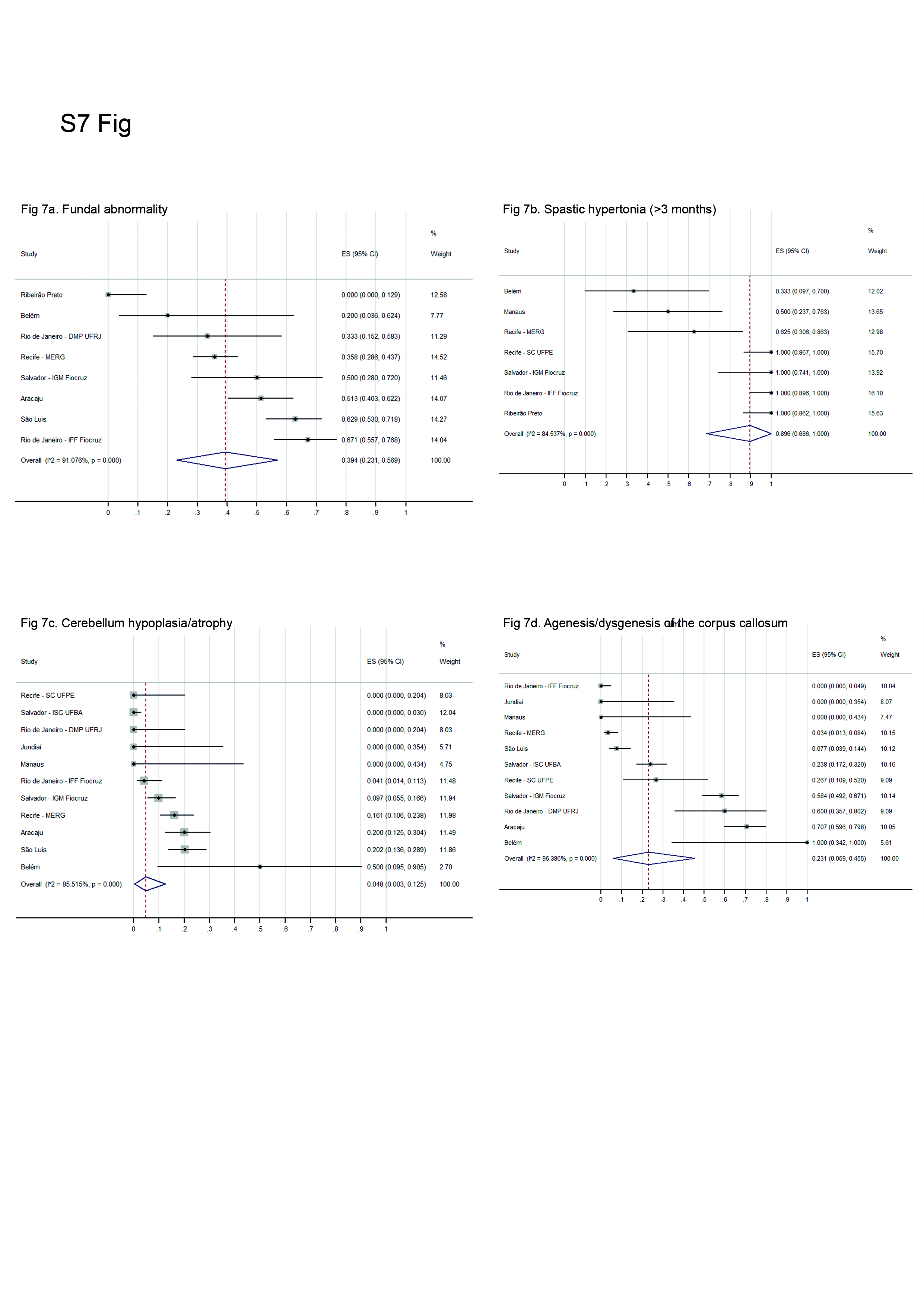

Supplement: S7 Fig — Frequency of fundal abnormality among the children (A) spastic hypertonia (B), cerebellum hypoplasia/atrophy (C) and agenesis/dysgenesis of the corpus callosum (D) in children with Zika-related microcephaly of the ZBC-Consortium. (TIF) [file pgph.0005425.s007.tif]
